# Efficient generation of Bessel-Gauss attosecond pulse trains via nonadiabatic phase-matched high-order harmonics

**DOI:** 10.1038/s41377-025-01845-7

**Published:** 2025-05-06

**Authors:** Mingxuan Li, Xiangyu Tang, Huiyong Wang, Jialong Li, Wentao Wang, Jiaao Cai, Jieda Zhang, Xinyue San, Xinning Zhao, Pan Ma, Sizuo Luo, Cheng Jin, Dajun Ding

**Affiliations:** 1 Institute of Atomic and Molecular Physics, Jilin University, 130012 Changchun, China; 2 Jilin Provincial Key Laboratory of Applied Atomic and Molecular Spectroscopy, Jilin University, 130012 Changchun, China; 3 Department of Applied Physics, Nanjing University of Science and Technology, 210094 Nanjing, China; 4 MIIT Key Laboratory of Semiconductor Microstructure and Quantum Sensing, Engineering Research Center of Semiconductor Device Optoelectronic Hybrid Integration in Jiangsu Province, Nanjing University of Science and Technology, 210094 Nanjing, China

**Keywords:** Nonlinear optics, Ultrafast photonics, High-harmonic generation

## Abstract

Generating Bessel-Gauss beams in the extreme ultraviolet (EUV) with attosecond pulse durations poses a significant challenge due to the limitations of conventional transmission optical components. Here, we propose a novel approach to produce such beams by inducing an annular EUV source through high-order harmonic generation (HHG) under nonadiabatic phase-matching conditions. The resulting light pulse maintains temporal coherence and manifests attosecond pulse trains as confirmed by the reconstruction of attosecond beating by interference of two-photon transitions (RABBIT) measurements. Macroscopic HHG calculations reproduce the measured spatiotemporal structures, demonstrating the plasma-induced spatial modulation on the formation of an annular source. Propagation simulations further confirm the feasibility of this approach for generating attosecond Bessel-Gauss beams, presenting exciting prospects for various applications in EUV photonics and attosecond science.

## Abstract (web summary)

Attosecond EUV Bessel-Gauss beam can now be generated through propagating an annular high harmonic source produced by the interaction of a strong laser with atomic gas.

## Graphical abstract

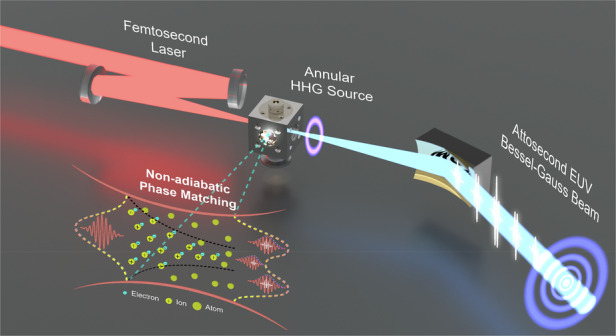

## Introduction

A laser beam passing through a narrow circular slit placed in the focal plane of a lens forms a diffraction-free beam, as proposed by Durnin et al.^1,2^, which is known as a Bessel-Gauss beam^3^ due to its spatial intensity distribution during propagation in the free field^4^. The Bessel-Gauss beam is nondiffracting in the zone of the conical superposition of plane wave vectors, with the extraordinary focusing and self-healing characteristics that have been used for laser ablation^5,6^, optics trapping^7^, microscopy and long-range sensing^8–10^, as well as quantum entanglement^11,12^. These applications span the frequency range from microwave to ultraviolet with various generation methods, such as the use of a circular slit^1^, axicon^13,14^, and holography^10,15,16^. However, the above methods have difficulties in extending the generation of the Bessel-Gauss beam into the EUV regime due to the absorption property of optical materials. The EUV Bessel-Gauss beam in attosecond duration holds significant potential to advance applications in coherent imaging, EUV photolithography, and attosecond science^17–19^.

High-order harmonic generation (HHG)^20,21^ is widely used as a light source for attosecond science^22–28^ and EUV imaging^29–31^, owing to its intrinsic attosecond temporal resolution and the broad bandwidth in the EUV regime. The spatiotemporal structure of HHG has been extensively investigated, revealing a wealth of phenomena, including the attosecond lighthouse effect^32,33^, quantum path interference^34–36^, and caustics^37^. Furthermore, structured near-infrared (NIR) laser beams have also been used to drive this nonlinear process, e.g., Bessel-Gauss^38–40^, truncated Bessel^41^, and annular beams^42^. Since the non-diffraction property of the Bessel-Gauss beam is not inherent, it cannot be directly transferred through the up-conversion process, making the generation of attosecond Bessel-Gauss beams in the EUV regime a challenging task. In addition, wavefront control of the fundamental laser with solid-based high harmonic^43^ has been used to produce the Bessel-like beam in the vacuum ultraviolet (VUV) regime. Furthermore, a transmission diffraction ring grating has been theoretically proposed to generate EUV Bessel-Gauss beams with a narrow bandwidth^18^. Recently, HHG in the nonadiabatic phase-matching regime, achieved when the ionization level exceeds the critical value^44,45^, where the laser intensity is high enough to suppress the potential barrier^46–48^ has attracted extensive attention due to its unique spatiotemporal behaviors^33,42,49–52^. This provides a feasible route to directly modulate the attosecond EUV source, allowing propagation effects presenting on the EUV beam.

In this work, we first show the unique and robust spatiotemporal behavior of HHG in the nonadiabatic phase-matching regime through experimental measurements. By combining theoretical simulations of the 3D propagation of the fundamental laser and high-harmonic fields in the gas medium, an intuitive interpretation of HHG behaviors is offered. The reshaping of the driving laser due to the plasma effect strongly modifies the spatial distribution of the harmonic source over tens of microns, acting like a manipulative native circular slit. Furthermore, propagation simulations based on Fresnel–Huygens diffraction verify the feasibility of utilizing this source to generate an attosecond EUV Bessel-Gauss beam. This unique spatial effect, robustly modulated by laser-gas interaction, paves the way for a new generation of non-diffraction attosecond beams, particularly suitable for EUV coherent imaging and photolithography, and provides a powerful new tool for attosecond methodology.

## Results

### Protocol for generating EUV Bessel-Gauss beam

**Fig. 1:**
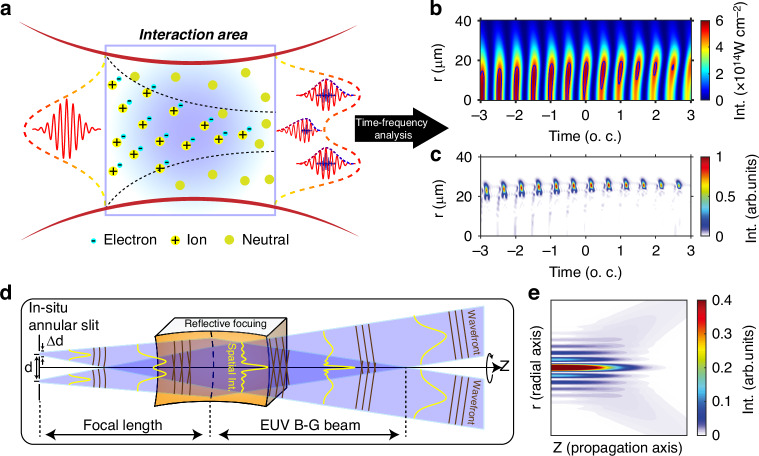
Formation of an attosecond Bessel-Gauss beam. **a** Illustration of the annular EUV emissions resulting from the reshaping of the intense femtosecond NIR laser after propagation and harmonic generation in the nonadiabatic phase-matching regime. **b** Spatiotemporal structure of the NIR laser field at the exit plane. The blue lines represent contours with a laser intensity of 5 × 10^14^ W cm^−2^. **c** Attosecond bursts observed at the exit plane of the interaction area. **d** The optical path for generating an attosecond EUV Bessel-Gauss beam using the annular EUV source. **e** Evolution of the Bessel-Gauss beam simulated for the annular source along the propagation axis

The method proposed by Durnin et al.^1,2^ inspired us to generate the annular EUV source by producing HHG within the nonadiabatic phase-matching regime, as shown in Fig. 1a (the setup shown in Supplementary Data). In such regime, the ionization level of atoms in the gas cell is much higher than the critical ionization *p*_*cr*_, defined by ${p}_{cr}={[({N}_{0}{r}_{0}{\lambda }_{1}^{2})/(2\pi \delta n)+1]}^{-1}$, where *N*_0_ is the atom density, *r*_0_ is the Bohr radius, the driving laser wavelength is defined as *λ*_1_, and *δn* is the refractive index difference between the fundamental laser and harmonic fields. Moreover, the phase-matching condition becomes space- and time-dependent after considering the full evolution of laser-atom interaction in the gas cell. In this procedure, the on-axis laser intensity of the simulated driving NIR is significantly reduced as a result of plasma dispersion-induced intensity decay, as illustrated in Fig. 1b. Consequently, the HHG emitted within this time-space window does not meet the phase-matching condition and gives a very low intensity. Simultaneously, the plasma dispersion effect shifts the peak laser intensity to off-axis positions for time larger than 0 o.c. (optical cycle), maintaining satisfactory phase-matching conditions for HHG signals in the off-axis region, as shown in Fig. 1c. The harmonic field propagates from the near-field to the far field, as calculated using Eq. (7), the volume integration of *rdr* causes the harmonic emissions on the axis at the exit of the gas medium to nearly vanish in the resulting Bessel-Gauss attosecond pulses.

This configuration leads to the formation of a cylindrically symmetric annular EUV source with a relatively flat wavefront profile, where the plasma effect acts as an in situ circular slit with a width of approximately several micrometers (μm) within a radius of tens of μm. This distinct EUV source serves as the basis for generating an attosecond Bessel-Gauss beam, following the optical paths outlined in Fig. 1d. A conically superpositioned plane wave can be generated using an annular source placed at the focal plane of a reflective EUV focusing mirror. Within this region, the beam’s transverse cross-section, symmetrically circular along the propagation axis, exhibits an intensity profile resembling the Bessel function (${J}_{0}^{2}$) modulated by a Gaussian profile dependent on propagation. Thus, the annular EUV source facilitates the formation of a Bessel-Gauss beam in the far field after passing the reflective EUV focusing mirrors. Consequently, the attosecond beam in this region exhibits the propagation properties of a Bessel-Gauss beam in the free space, as indicated in Fig. 1e.

### Spatial-spectral distribution of HHG in the nonadiabatic regime

**Fig. 2:**
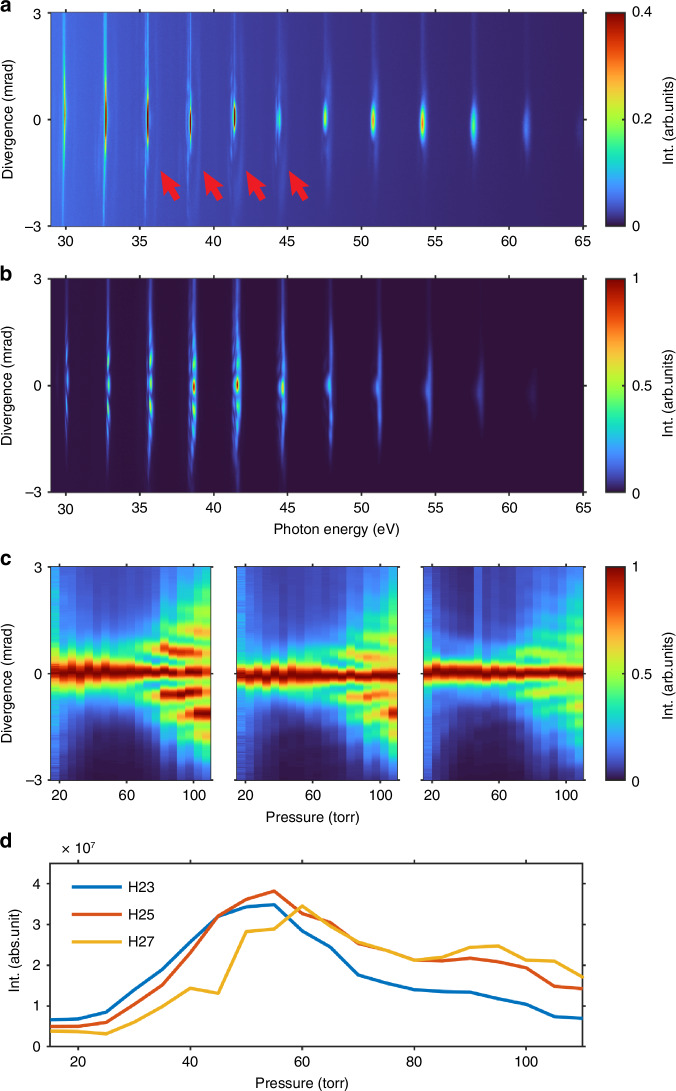
Spatial-spectral structure of HHG from Ar gas. **a**, **b** Measured spatial distributions of HHG spectra from Ar at pressures of 20 Torr and 80 Torr in the nonadiabatic phase-matching regime at the intensity of 5 × 10^14^ W cm^−2^. The signals contributed by the long trajectories are indicated by red arrows. **c** Spatial distributions of harmonic signals for H23, H25, and H27 as a function of gas pressure. The signals are integrated over an energy range of 2 eV and normalized separately. **d** Gas pressure-dependent harmonics intensities for H23, H25, and H27

To demonstrate the wide applicability of the protocol and provide a broad spectral regime in the EUV Bessel-Gauss beam generation, we investigated the spatial distribution of HHG from argon (Ar) and neon (Ne) gases under nonadiabatic phase-matching conditions, covering energy ranges of 29–60 eV and 50–72 eV, respectively. Figure 2 illustrates the HHG spectra obtained from the Ar gas, ranging from 19th harmonic (H19) to H39, backing pressures increasing from 15 to 110 Torr, with a laser intensity of 5 × 10^14^ W cm^−2^, causing the initial ionization level much higher than the critical value (≈4% for argon at 800 nm). The spectra obtained under pressures of 20 and 80 Torr are presented in Fig. 2a, b, respectively. At low gas pressures, the HHG spectra exhibit a typical spatial distribution without noticeable diffraction patterns, and there is a weak contribution from the long trajectory^53^ as indicated by the red arrows. However, as the gas pressure increases, pronounced stripes appear in the off-axis spatial distribution. The same trend is observed in our simulations, as shown in Supplementary Data. In addition, the HHG signals undergo a significant blue shift due to self-phase modulation of the driving laser^54^, and no contribution from long trajectories has been observed under these conditions. This can be explained by the fact that phase mismatch is more pronounced for high harmonics from long electron trajectories, which cannot be compensated by varying the gas pressure, and thus unfavorable phase-matching conditions are presented^46,51,55^.

Furthermore, Fig. 2c, d displays the pressure-dependent divergences and brightness for H23, H25, and H27. At high pressures, the off-axis spatial distribution becomes more significant, with the spacing of the stripes showing a noticeable dependence on the gas pressure and harmonic order. The relative intensity ratio between the 80 Torr and the optimal pressure (55 or 60 Torr) is ~40%, 55%, and 62% for H23, H25, and H27, respectively. The increasing plasma density affects the spatiotemporal evolution of the driving laser in the gas medium, influencing harmonic generation in two ways. First, the harmonic source is predominantly modulated into an annular shape. Second, the phase-matched region shifts further off-axis, facilitating the effective build-up of harmonics. The radius of the annular source increases as the plasma density in the center of the laser beam increases, consequently narrowing the spacing between stripes. This phenomenon has been replicated in our simulations, and the results are provided in Supplementary Data.

### Macroscopic propagation effect

**Fig. 3:**
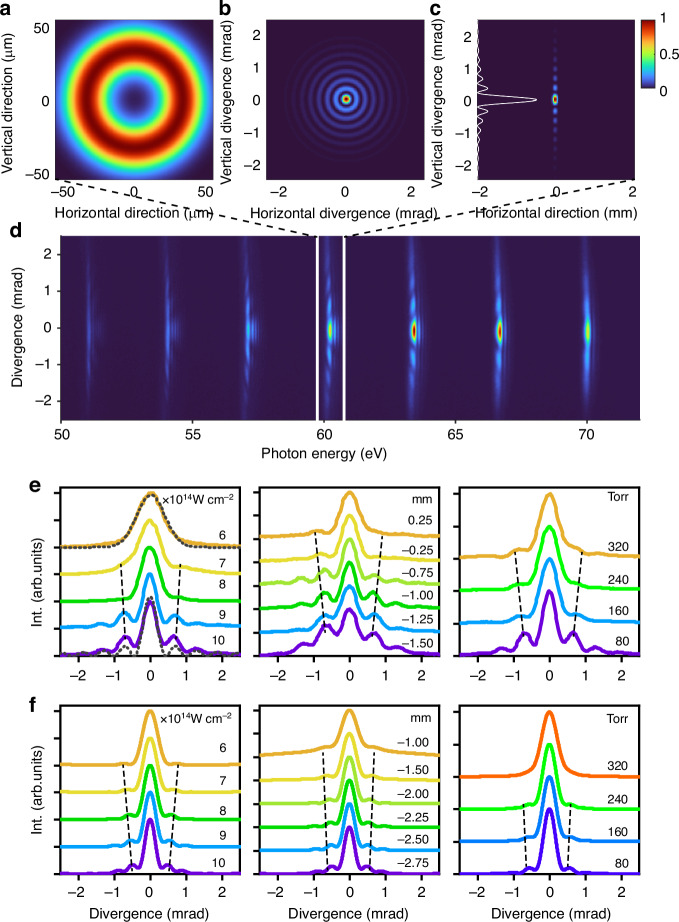
Macroscopic propagation effect on spatial profiles. **a** Annular EUV Source (H39) in ray tracing simulation. **b** Free propagation imaging to the front of the grating. **c** Captured spectrum located in the focal plane of the grating. **d** Experimentally measured full spectrum of HHG from Ne gas with a pressure of 80 Torr and a target position of −1 mm under a laser intensity of 10 × 10^14^ W cm^−2^. **e** The divergence of H39 in experiments was obtained by: (left) varying the laser intensity at a gas pressure of 80 Torr and a target position of −1 mm, with the dashed black curve representing the Bessel distribution; (middle) varying the target position, with a pressure of 80 Torr and a laser intensity of 10 × 10^14^ W cm^−2^; (right) varying gas pressure, with a laser intensity of 10 × 10^14^ W cm^−2^ and a target position of −1 mm. **f** Theoretical simulated divergence of H39 obtained under the same experimental conditions. The black dashed lines guide the location of the first satellite peak

To clarify the effect of flat-field gratings on detection, we simulate the propagation of an annular EUV source (H39) using ray tracing. The important spatial distributions from beam tracing for this annular source, its free propagation before reaching the grating, and its distribution at the focal plane after grating, are shown in Fig. 3a–c. The beam exhibits a Bessel-like distribution after free propagation, as shown in Fig. 3b. After passing through the grating, the horizontal dimension corresponding to the energy distribution of photons is compressed, while the vertical divergence remains unchanged, consistent with the measured harmonic spectra. It gives an intensity distribution which can be described with the Bessel function (${J}_{0}^{2}$), as shown in Fig. 3c.

To better understand the influence of plasma effect and interaction geometry on the spatial distribution of HHG spectra under nonadiabatic conditions, and to verify the universality of the generating medium for producing the EUV Bessel-Gauss beam, we conducted measurements of spatially resolved H33 to H45 generated from Ne gas. The full spectrum is presented in Fig. 3d. We observed similar spatial distributions compared to those obtained from Ar gas, as shown in Fig. 2b. The divergence profiles of H39, modulated by laser intensity, target position, and gas pressure, are shown in Fig. 3e. At low laser intensities, the divergence shows a Gaussian profile with no discernible diffraction structure. However, as the laser intensity increases, particularly surpassing the Ne’s barrier-suppression intensity (~8.6 × 10^14^ W cm^−2^), the off-axis signal gradually increases while the width of the central peak decreases. At the same time, as the laser intensity increases to 10 × 10^14^ W cm^−2^, the observed beam profile transitions to a Bessel-like distribution, as highlighted in comparison to the black dashed Bessel distribution in the left panel of Fig. 3e.

Furthermore, when the gas target was placed far in front of the focus (−1.5 mm), only a vague diffraction structure was observed. Moving the focus closer to the center of the gas cell (−0.75 mm) intensified the diffraction signal. Within a range near the focal point, we observed significant modulation of the degree of diffraction and the extent of divergence. Finally, after passing through the focal point (at 0.25 mm), the spatial spectrum became essentially smooth. However, the phenomenon of gas pressure change significantly differs from the behavior observed in Ar gas, as shown in Fig. 2c. In the case of Ne, an increase in the gas density significantly depleted the off-axis part of the intense NIR laser, causing the harmonic emission to relocate to the on-axis position in the near-field, which leads to a Gaussian profile at high gas pressure.

To interpret these experimental findings, we conducted a comprehensive quasi-three-dimensional (3D) simulation, accounting for macroscopic propagation effects within the dense gas and plasma environment. This simulation sheds light on the mechanisms underlying the EUV Bessel-Gauss beam formation process. In Fig. 3f, we present the simulated divergence for comparison with the measured spectra under identical experimental conditions. Notably, we observe a similar dependence of the simulated spatial profiles of H39 on laser intensity, target position, and gas pressure compared to the observations. Given that the mechanism of creating an annular EUV source in the gas medium remains consistent for all harmonics in the plateau region, we selected H39 for comparison with measurements to achieve the best agreement. The appearance conditions and the shifting trends of the subpeaks (guided by the black dashed lines in Fig. 3e, f) exhibit remarkable agreement between experiment and theory. These results imply that the spatial-spectral structure of HHG generated in the nonadiabatic phase-matching regime can be significantly altered by the laser intensity and interaction geometry, thus demonstrating that the modifications in the propagation effects of the fundamental laser and high-harmonic field in the gas medium contribute to the observed changes in spatial structure. Furthermore, the evolution of the near-field of the light source can provide additional confirmation of this notion (as detailed in the Supplementary Data).

### Spatiotemporal analysis of the attosecond Bessel-Gauss beam

**Fig. 4:**
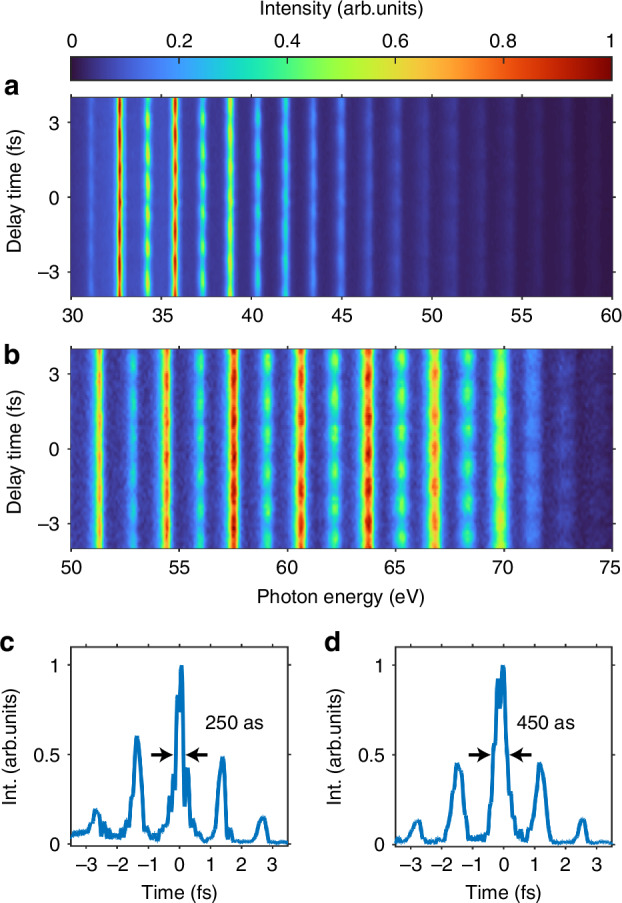
Temporal reconstruction of APT with Ar and Ne Sources. **a**, **b** RABBIT spectra of Ar and Ne sources in nonadiabatic phase-matching regime. **c**, **d** Temporal structure of APT reconstructed using the RABBIT spectra from Ar and Ne sources by employing the ePIE method

We experimentally scanned the RABBIT traces to validate the temporal coherence of the Bessel-Gauss HHG source. The relative spectral phase between neighboring harmonics can be extracted from the delay-dependent amplitude of sidebands (S_SB_) signals, which generated from the interference of two two-photon transition paths^56^. The intensity of the sidebands oscillates according to

1 $${S}_{{\rm{SB}}}={\mathcal{A}}+{\mathcal{B}}\cos [2{\omega }_{0}(\tau -{\tau }_{{\rm{atom}}}-{\tau }_{{\rm{XUV}}})]$$

where A and B are constant, *ω*_0_ is the angular frequency of the fundamental laser field, *τ* is the time delay between attosecond pulse trains (APT) and NIR pulses, *τ*_atom_ is the atomic two-photon ionization time delay, and *τ*_XUV_ is the group delay of the attosecond pulses^57^. Using helium (He) as the target gas, the measured RABBIT spectra for HHG from Ar and Ne are presented in Fig. 4a, b. Reconstruction was performed by optimizing the fit to the measured RABBIT spectra using the extended ptychographic iterative engine (ePIE)^58^. The retrieved atto-burst durations in the APT have full-width-at-half-maximum (FWHM) values of ~250 as and 450 as, respectively, as shown in Fig. 4c, d (see additional details in the Supplementary Data).

**Fig. 5:**
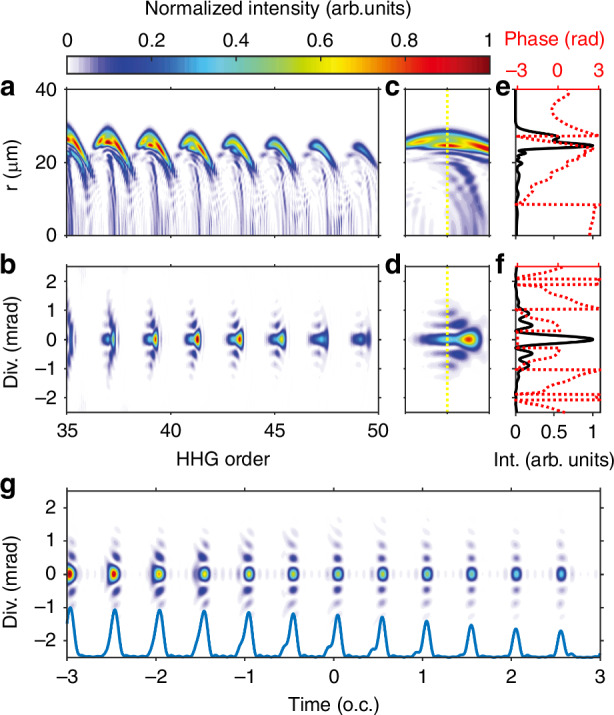
Spatial-spectral and temporal structure analysis of HHG source. **a**, **b** Simulated full spectra of Ne in the near and far fields, with a pressure of 80 Torr in the laser-gas interaction region, a gas position of −1 mm and a laser intensity of 10 × 10^14^ W cm^−2^. **c**, **d** The zoomed-in near and far fields spatial distribution of H39. **e**, **f** Intensity and phase distribution for the selected energy slice marked in (**c**, **d**). **g** Spatiotemporal structure of H35-H47 obtained by fast Fourier transform and the synthesized attosecond pulse trains by integrating along the spatial direction is shown in the blue line

Theoretically, we can comprehensively analyze the generation mechanism and characterize the spatiotemporal structure of attosecond Bessel-Gauss pulse trains. Figure 5 illustrates the simulated spatiotemporal distributions of H35-H47 from Ne. In Fig. 5a, we observe that the harmonics emitted in the near-field (at the exit plane of the gas medium) exhibit significant off-axis components at approximately *r* = 20 μm for H35-H47. This annular EUV source with a flat phase evolves into a Bessel-like diffraction pattern in the far field, as shown in Fig. 5b. This behavior is more evident in the zoomed-in patterns around H39, as depicted in Fig. 5c, d. The spatial phase in the off-axis region for H39 slowly varies along the radial (Fig. 5e), while different off-axis parts in the far field exhibit almost *π* phase jumps for the divergence profile (Fig. 5f).

Furthermore, we performed an analysis (detailed in Supplementary Data) to trace the correlation between the spatial structures of the HHG source in the near and far fields. The EUV pulses in the temporal domain, synthesized by integrating H35-H47, maintain a Bessel-like intensity profile along the radial direction in the far field (Fig. 5g). The duration of each burst in the APT is ~350 as. These observations demonstrate the coexistence of spatial and temporal coherence in the HHG source and its potential for compression into an APT.

### Propagation of the attosecond Bessel-Gauss beam

**Fig. 6:**
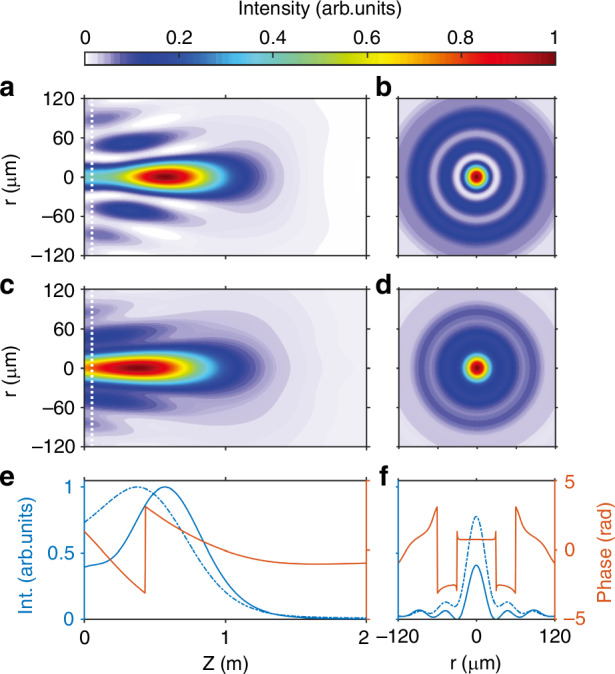
Numerical simulation of the spatial distribution of the EUV source. **a** EUV Bessel-Gauss beam produced by the annular EUV source using the energy slice on H39. **b** Radial distribution corresponding to that from the white dotted line in (**a**). **c** Attosecond EUV Bessel-Gauss beam generated by the annular EUV source using H35-H47. **d** Radial section at the white dotted line in (**c**). **e** On-axis intensity and phase distribution in (**a**, **c**). The solid lines represent the energy slices on H39, and the dashed lines are the energy integrals of H35-H47. **f** The intensity and phase derived from the radial profiles for the distributions of (**b**, **d**)

Moreover, the propagation of the Bessel-Gauss EUV beam was simulated by using the Fresnel–Huygens integral, which allows us to acquire both the intensity and phase of the beam profile. The EUV source was placed in the focal plane of the reflective focus mirror, allowing free propagation of the EUV beams for a slice at H39 (Fig. 5c) and the integrated range of H35-H47 (Fig. 5a). The simulated results are presented in Fig. 6. In the case of both monochromatic and synthetic attosecond EUV light, the intensity distribution tends to oscillate on the cross-section and gradually diminishes as the beam propagates along the *z* axis. At a fixed position *z*, the intensity distribution follows a zero-order Bessel distribution (${J}_{0}^{2}$) in Fig. 6a–d. Meanwhile, a phase difference close to *π* occurs between adjacent peaks in the radial direction, as shown in Fig. 6e, f. This finding aligns with the typical features of the classical Bessel-Gauss beam^59^. Notably, it has a large depth-of-focus benefit from the propagation characteristics of the Bessel-Gauss beam. A similar propagation analysis was conducted for argon at 10 Torr, with results provided in Supplementary Data. These findings demonstrate the feasibility of generating an attosecond EUV Bessel-Gauss beam through the proposed high-harmonic generation methodology.

## Discussion

We demonstrated a new method for producing an attosecond Bessel-Gauss beam in the EUV regime. Our approach involves creating an EUV annular source by controlling the spatial-related harmonic generation process through reshaping the intense NIR laser during propagation in a gas medium. This occurs under conditions where the ionization level significantly exceeds the critical ionization threshold, and the gas pressure is relatively high. The excellent agreement between the experimental results and quasi-3D macroscopic phase-matching and propagation simulations supports the generation of an EUV-synthesized APT with a Bessel-Gauss spatial distribution. To further advance our research, we utilize the Huygens–Fresnel integral to demonstrate the formation of an EUV Bessel-Gauss beam through the propagation of this annular source. This groundbreaking method, which uses HHG in the nonadiabatic regime, opens up exciting possibilities for manipulating EUV beams. The unique nondiffracting properties of the attosecond EUV Bessel-Gauss beam along the propagation direction hold great promise for various applications in EUV photonics and attosecond science.

## Methods

The Bessel-Gauss attosecond pulse trains are generated by focusing a 35 fs laser pulse with a central wavelength of 800 nm into the Ar and Ne gas cell. To filter out the fundamental laser, the HHG radiation and the driving laser pass through a 500 nm thick Al foil. The produced HHG is then focused by an Au-coated toroidal mirror and sent into a slit-less spectrometer, an EUV charge-coupled device camera (Princeton Instruments, PIXIS-XO: 400B) captures the spatial-spectral distributions of the HHG after passing a flat-field concave grating (Hitachi, 001-0660, 1200 grooves mm^−1^). For RABBIT measurements, the photoelectron signal is recorded as a function of the delay between the APT and the probe NIR laser by using a high-energy resolution electron spectrometer, the parameters of the delay scan for each RABBIT measurement are 150 as step size, 8 fs total range and sub-30 as time jitter^26^. more details can be seen in the Supplementary Data.

In the simulations, quasi-three-dimensional (3D) Maxwell wave equations for the fundamental laser and high-harmonic field are solved in cylindrical coordinates. The evolution of the fundamental field is described by^60^

2 $${\nabla }^{2}{E}_{1}(r,z,t)-\frac{1}{{c}^{2}}\frac{{\partial }^{2}{E}_{1}(r,z,t)}{\partial {t}^{2}}={\mu }_{0}\frac{\partial {J}_{{\rm{abs}}}(r,z,t)}{\partial t}+\frac{{\omega }_{0}^{2}}{{c}^{2}}(1-{\eta }_{\text{eff}\,}^{2}){E}_{1}(r,z,t)$$

where *E*_1_ is the transverse electric field with central frequency *ω*_0_. ${\nabla }^{2}={\nabla }_{\perp }^{2}+{\partial }^{2}/\partial {z}^{2}$ is Laplacian operator in cylindrical coordinates. The effective refractive index is

3 $${\eta }_{{\rm{eff}}}(r,z,t)={\eta }_{0}(r,z,t)+{\eta }_{2}I(r,z,t)-\frac{{\omega }_{p}^{2}(r,z,t)}{2{\omega }_{0}^{2}}$$

Here, the linear term *η*_0_ = 1 + *δ*_1_ − *iβ*_1_ accounts for refraction (*δ*_1_) and absorption (*β*_1_) by the neutral atoms, the second term describes the optical Kerr nonlinearity which depends on the instantaneous laser intensity *I*(*t*), and the third term contains the plasma frequency ${\omega }_{p}={[{e}^{2}{n}_{e}(t)/({\varepsilon }_{0}{m}_{e})]}^{1/2}$, where *m*_*e*_ and *e* are the mass and charge of an electron, respectively, and *n*_*e*_(*t*) is time-dependent density of free electron. The absorption due to ionization of the gas medium is given by

4 $${J}_{{\rm{abs}}}(t)=\frac{\gamma (t){n}_{0}(t){I}_{p}{E}_{1}(t)}{| {E}_{1}(t){| }^{2}}$$

where *γ*(*t*) is the ionization rate, *n*_0_(*t*) is the density of neutral atoms, and *I*_*p*_ is the ionization potential. Ionization rates involved in Eq. (4) is calculated using the Ammosov–Delone–Krainov (ADK) theory. The fundamental laser field is assumed to be Gaussian both in space and time at the entrance of the medium, and the gas pressure is a constant within the medium.

The 3D propagation equation of the high-harmonic field is^60^

5 $${\nabla }^{2}{E}_{h}(r,z,t)-\frac{1}{{c}^{2}}\frac{{\partial }^{2}{E}_{h}(r,z,t)}{\partial {t}^{2}}={\mu }_{0}\frac{{\partial }^{2}P(r,z,t)}{\partial {t}^{2}}$$

where *P*(*r*, *z*, *t*) is the polarization depending on the applied fundamental field *E*_1_(*r*, *z*, *t*). Here, the free-electron dispersion is neglected because the frequencies of high harmonics are much higher than the plasma frequency. In general, the polarization *P*(*r*, *z*, *t*) is separated into linear and nonlinear components, and the linear susceptibility *χ* includes both linear dispersion and absorption effects of the harmonics. The nonlinear polarization term *P*_*nl*_(*r*, *z*, *t*) can be expressed as

6 $${P}_{nl}(r,z,t)=[{n}_{0}-{n}_{e}(r,z,t)]D(r,z,t)$$

where *n*_0_ − *n*_*e*_(*t*) gives the density of the remaining neutral atoms, and *D*(*r*, *z*, *t*) is the single-atom-induced dipole moment. Note that Eqs. (2) and (5) are solved by using the Crank-Nicholson routine in the frequency domain.

Once the harmonic field at the exit face (near-field) of the medium is computed, its propagation in free space can be obtained from near-field harmonics through a Hankel transformation. In the paraxial approximation, far-field harmonics can be calculated as^60^

7 $${E}_{h}^{f}({r}_{f},{z}_{f},\omega )=ik\mathop{\int}\nolimits_{0}^{\infty }\frac{{E}_{h}^{n}(r,{z}_{n},\omega )}{{z}_{f}-{z}_{n}}\times {J}_{0}\left(\frac{kr{r}_{f}}{{z}_{f}-{z}_{n}}\right)\exp \left[-\frac{ik({r}^{2}+{r}_{f}^{2})}{2({z}_{f}-{z}_{n})}\right]rdr$$

where *J*_0_ is the zeroth-order Bessel function, the wave vector *k* is given by *k* = *ω*/*c* with the harmonic frequency *ω*. In Eq. (7), the positions of near and far fields are given by *z*_*n*_ and *z*_*f*_, respectively. ${E}_{h}^{n}(r,{z}_{n},\omega )$ is the near-field harmonic; *r* and *r*_*f*_ are the transverse coordinates in the near and far fields, respectively.

## Supplementary information

Supplementary Information
