# Supplementary material for: Efficient generation of Bessel-Gauss attosecond pulse trains via nonadiabatic phase-matched high-order harmonics
Source: Light Sci Appl. 2025 May 6;14:181. doi: 10.1038/s41377-025-01845-7 (PMC12053678; doi:10.1038/s41377-025-01845-7)
Supplement: Supplementary file 1 — Supplementary Information [file 41377_2025_1845_MOESM1_ESM.pdf]

Supplementary Information for Efficient generation of Bessel-Gauss attosecond pulse trains via nonadiabatic phase-matched high-order harmonics

Mingxuan Li[1,2†], Xiangyu Tang[3†], Huiyong Wang[1,2], Jialong Li[1,2], Wentao Wang[1,2], Jiaao Cai[1,2], Jieda Zhang[1,2], Xinyue San[1,2], Xinning Zhao[1,2], Pan Ma[1,2], Sizuo Luo[1,2*], Cheng Jin[3,4*], Dajun Ding[1,2*]

[1]Institute of Atomic and Molecular Physics, Jilin University, Changchun, 130012, China.
[2]Jilin Provincial Key Laboratory of Applied Atomic and Molecular Spectroscopy, Jilin University, Changchun, 130012, China.
[3]Department of Applied Physics, Nanjing University of Science and Technology, Nanjing, 210094, China.
[4]MIIT Key Laboratory of Semiconductor Microstructure and Quantum Sensing, Engineering Research Center of Semiconductor Device Optoelectronic Hybrid Integration in Jiangsu Province, Nanjing University of Science and Technology, Nanjing, 210094, China.

*Corresponding author(s). E-mail(s): luosz@jlu.edu.cn; cjin@njust.edu.cn; dajund@jlu.edu.cn;
†These authors contributed equally to this work.

1 Experiment setup

To generate Bessel-Gauss attosecond pulse trains, the femtosecond laser was focused into a custom designed gas cell containing Ar or Ne gas, by a spherical mirror (Newport, focal length = 500 mm, with an Ag-coating), as illustrated in Fig. S1a. The gas cell consists of a 3 mm nickel tube and an auxiliary unit connected to a scroll pump (Edward, XDS 35i). During the measurements, the position of the gas cell

can be adjusted precisely using a three-dimensional stage with an accuracy of 10 μm along three axes. The produced attosecond EUV annular beam was focused through a gold-coated toroidal mirror, and then, enters a slit-less EUV spectrometer for the detection of spatial diffraction without obstruction. In this setup, the EUV spectrum is spatially separated by a Hitachi flat-field concave grating mounted on a five-axis tilt aligner (Newport, 8081M-UHV) and captured by the EUV camera (Princeton Instruments, PIXIS-XO: 400B). It's worth noting that the EUV spectrometer exhibits a high energy resolution, surpassing $E/\Delta E > 1200$ in the wavelength range of 5–25 nm. This configuration ensures remarkable energy resolution and spatial detection capabilities, providing precise spatial-spectral spectrum analysis of HHG radiation. Fig. S1b illustrates the measured focal profile of the driving laser at the gas cell by CCD 1. Additionally, Fig. S1c showcases a distinct diffraction pattern corresponding to H25 from argon gas measued by the EUV camera.

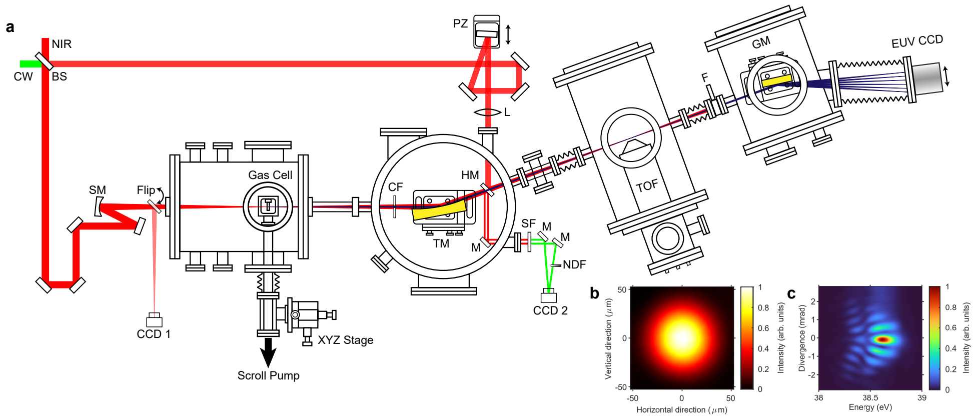

Fig. S 1 | **Schematic diagram of the experimental setup. a,** The optical path and components. CW: Continuous Wave Laser, BS: Beam Splitter, SM: Spherical Mirror, CCD: Charge Coupled Device, CF: Coaxial Foil, TM: Toroidal Mirror, HM: Holey Mirror, SF: Spatial Filter, NDF: Neutral Density Filter, TOF: Time of Flight, F: Al foil, GM: Grating Mirror, PS: Piezo Stage. **b,** NIR focus spot at the harmonic generation gas cell. **c,** Spatial and spectrum distribution of H25 from argon.

Additionally, we reconstructed the pulse duration of APT and characterized the temporal coherence of HHG from Ar and Ne gases in the nonadiabatic phase-matching regime through RABBIT measurements. These measurements involved collecting delay-dependent photoelectrons using a high-energy resolution time-of-flight spectrometer (Stefan, ETF-11). To control the delay between the EUV pump and NIR probe beams with attosecond precision, the stabilization of the Mach–Zehnder interferometer is achieved by locking the piezoelectric translation stage (Physik Instrumente, P-752.1CD) using feedback signals retrieved from the interference fringes of the co-propagating 532 nm laser. The jitter between two beams is approximately 30 attoseconds within 12 hours in this active stabilization system.

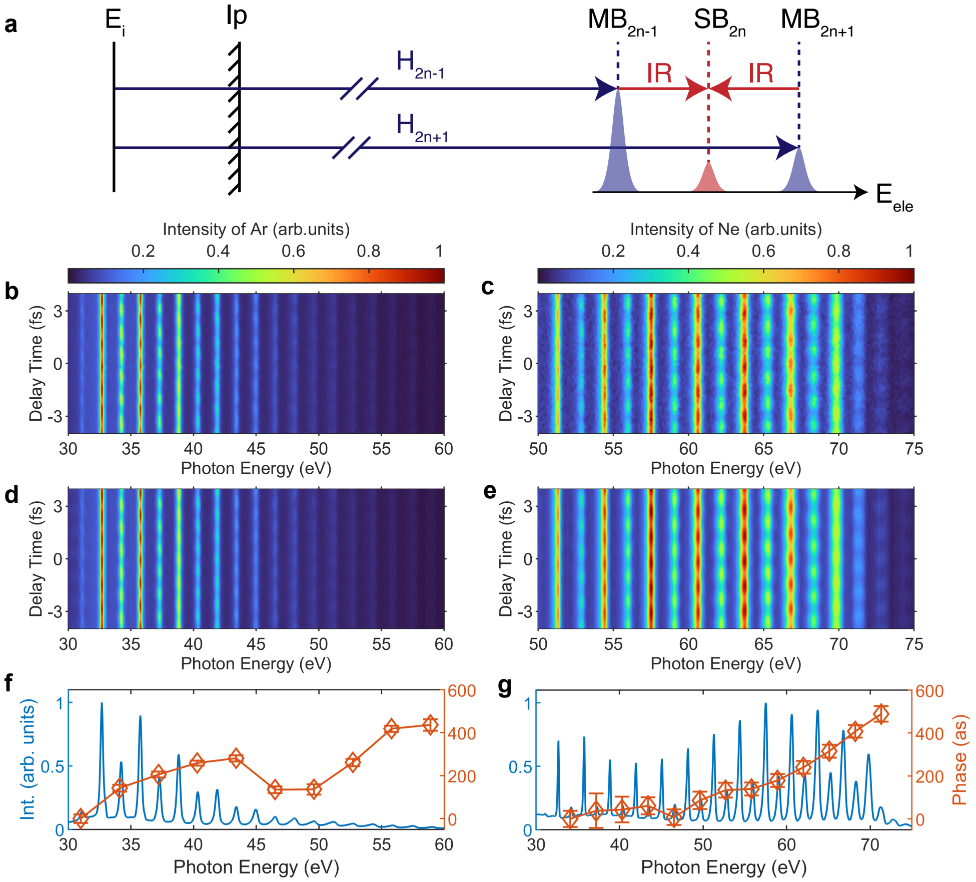

Fig. S 2 | Related RABBBIT measurements and analysis. a, Schematic of the two-photon path interference in RABBIT method. **b,c** Experimental spectra, **d,e** reconstructed spectra, and **f,g** integral signal with extracted relative phase of each SB for argon and neon, respectively.

The schematic of RABBIT is shown in Fig. S2a. The initial bounded electron absorb one EUV photon ($H_{2n\pm1}$) and populated to the mainband ($MB_{2n\pm1}$) in continuum state. Under a perturbed NIR probe field, two adjacent MBs will produce a sideband because of the interference from the absorption and emission of one NIR photon each. In Fig. S2b-c are the experimental measured spectra, and (d) and (e) are the reconstructed spectra. We have also extracted the attosecond chirp for the experimental HHG spectra, (f) for argon and (g) for neon. The corresponding attosecond chirp of the harmonics has an important influence on extracting the actual duration of APT. Apart from the fact that the spectrum of argon has a special structure due to the presence of CM intervals, the slope of attochirp in argon is smaller compared to that of neon for the main part of the EUV, which may have led to a more concentrated emission of the APT of Ar.

2 Theoretical verification of spatial and spectral distributions in HHG from argon gas

Theoretically, we performed an extensive simulation of HHG originating from Ar gas. This was accomplished by solving the three-dimensional Maxwell's equations for both the fundamental laser and the generated high-harmonic field while taking into account variation of gas pressure. Further details regarding this simulation can be found in the dedicated "Numerical Simulation" section. Fig. S3a presents the simulated spatial-spectral structure of HHG generated from Ar gas under a laser intensity of 2.3×10^{14} W cm^{-2}, at a gas pressure of 140 Torr. The simulation results reveal a distinct Bessel-like profile when the harmonics are situated between 11th and 19th orders. Additionally, we constructed integral divergence profiles for harmonics of different orders, as depicted in Fig. S3b. It becomes evident that as the harmonic order increases, the spacing between fringes decreases, aligning perfectly with our experimental observations. Meanwhile, we extracted and presented divergence profiles of the 13th harmonic (H13) at varying gas pressures, as illustrated in Fig. S3c. As the gas pressure increases from 40 Torr to 140 Torr, the spacing between fringes narrows, effectively corroborating the transition observed in our experimental findings showcased in the main text.

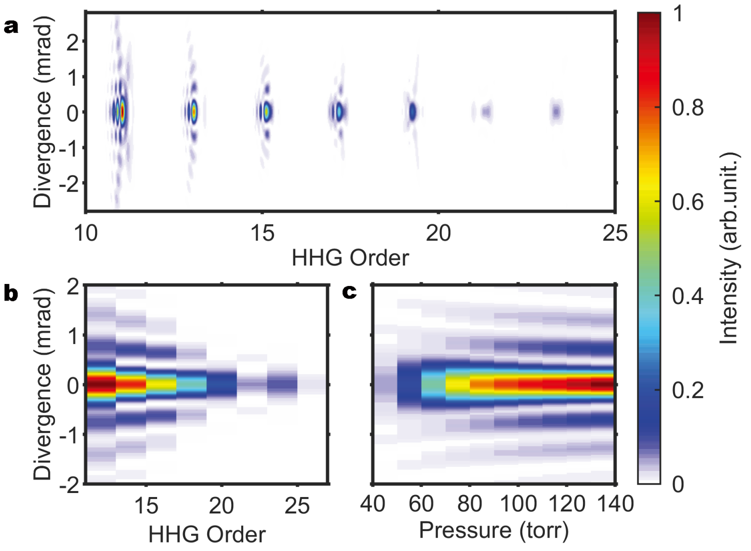

Fig. S 3 | **Spatial-spectral structure of HHG from argon in simulation. a,** Spatial spectrum of argon at a pressure of 140 Torr under the laser intensity of 2.3×10^{14} W cm^{-2}. **b,** The order dependence of spatial divergence. **c,** Pressure dependence of spatial divergence of H13.

As shown in Fig. S4, there is a significant difference in the spatial-spectral configuration for different pressure under the same laser intensity. In the low-pressure interval, the near- and far-field spectra exhibit a Gaussian distribution spatially, and as the gas

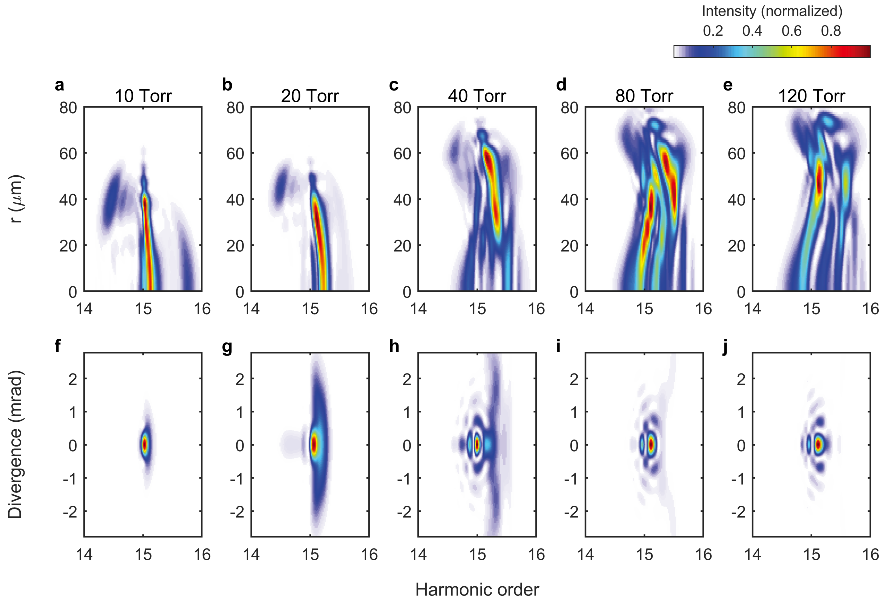

Fig. S 4 | Spatial-spectral analysis of HHG from argon in simulation. a-e, Near field structure of H15 in argon at a pressure of 10, 20, 40, 80, and 120 Torr with laser intensity of 2.3×10^{14} W cm^{-2}. **f-j,** Far field spectra with the same presure.

pressure increases, the near-field harmonic on-axis intensity decreases and the off-axis intensity increases, while the far-field also transforms to a Bessel-like distribution.

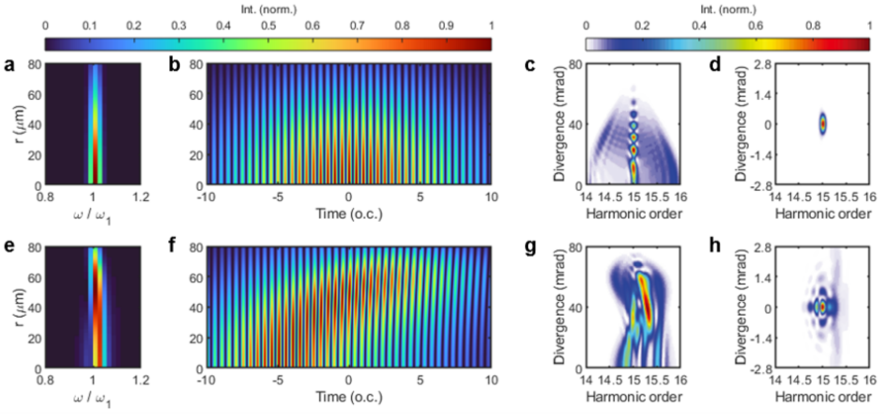

Fig. S 5 | Simulated spatial intensity of fundamental laser and harmonic source. a, Spatial spectrum and **b,** spatio-temporal intensity of fundamental laser at the entrance of argon target. Induced single-atom harmonic source of H15 in the **c,** near field, and **d,** far field. **e-h,** The same as **a-d** except the position locates at the exit of target. The gas pressure is 40 Torr.

The harmonic frequency shift along the radial position in the near-field results from the blue shift of the driving laser. When propagating in a highly ionized medium, the plasma modulates the electric field, which corresponds to create a positive chirp in the driving laser that depends on the plasma density, as shown in Fig. S5f. The ionization level is higher when the axis is closer to the axis, so the electric field near the axis is more chirped, and consequently, the generated harmonic photon energy is more shifted. The photon spectrum that exists between the 15th and 16th orders without stripe is caused by the interference of short and long trajectories driven by a chirped laser. To clarify this, we first calculate the single-atom harmonic source using spatio-temporal driving laser fields at the exit of the gas medium, as shown in Fig. S5g, eliminating the effects of macroscopic propagation. This approach includes both harmonic emissions from short and long trajectories. One can observe the variations in the split structure along the radial position, which coincide with the chirp of the fundamental laser. These harmonics are considered to be near-field ones, which then propagate to the far field. The results in the far field are presented in Fig. S5h, resembling the non-integer harmonic structure shown in Fig. S4h.

3 Reshaping of NIR laser beams in dense gas mediums

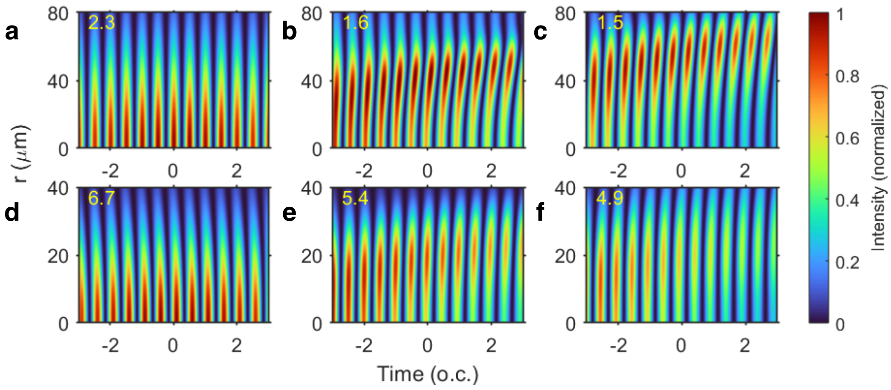

Fig. S 6 | Spatio-temporal structure of NIR field in the gas medium in simulation. Reshaping of NIR field during propagation in argon at **a,** entrance, **b,** middle, and **c,** exit. Each of them is normalized to its maximum value as labeled by red color and the unit is 10^{14} W cm^{-2}, respectively. **d-f,** Same as **a-c** but in neon.

As the tightly focused NIR laser beam travels through a dense gas medium, it induces the formation of a plasma along its path. This plasma formation initiates a simultaneous cascade of effects that transform the characteristics of the NIR beam. These effects encompass non-uniform reductions in intensity both parallel and perpendicular to the beam's propagation axis due to plasma-induced defocusing, changes in the laser field's shape, the emergence of a longitudinal gradient in gas ionization, and other related factors. The interplay between the plasma and the neutral gas leads

to alterations in the spatiotemporal structure of the NIR field as it evolves through propagation. This results in a phenomenon where the NIR field's shape is reshaped at various planes within the gas cell. The corresponding NIR pulses are shown in Fig. S6. The results presented in Fig. S6 show that as the driving laser field propagates through both Ar and Ne gases, it experiences significant spatial modulation. Consequently, an off-axis annular distribution of intensity becomes evident when the laser is propagated to the middle (see Fig. S6b and e) and the exit of the gas medium (see Fig. S6c and f).

The evolution of the driving laser is highly sensitive to boundary conditions, especially given its strongly non-linear nature. As illustrated in Fig. S7, the spatiotemporal intensity of the driving laser at the exit of the gas target varies with changes in gas pressure. Despite having identical initial laser fields, the laser profiles exhibit significant differences in both space and time after propagating through the gas medium.

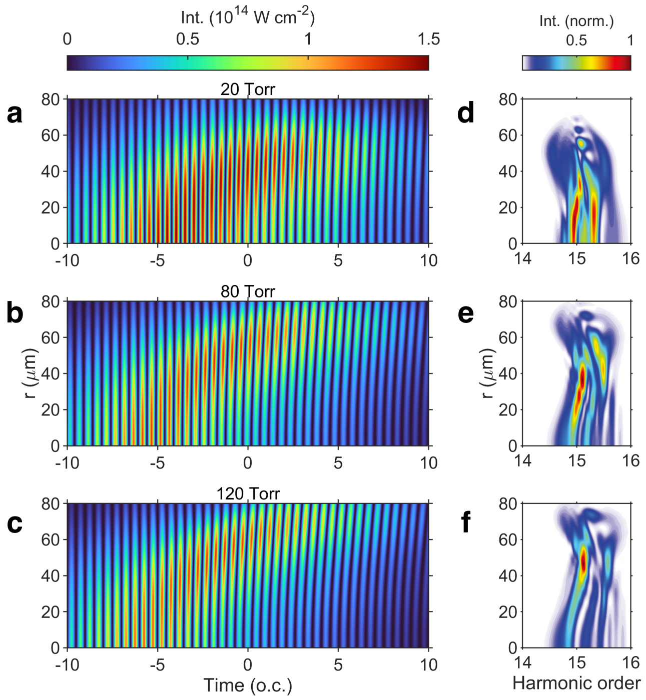

Fig. S 7 | Simulated propagated fundamental laser and induced harmonic source. a-c, Spatio-temporal intensity of propagated fundamental laser at the exit of argon target under different gas pressures. **e-f,** The corresponding induced single-atom harmonic source of H15.

Such variations in the fundamental laser properties influence the high-order harmonic generation (HHG) process through two primary mechanisms: the harmonic source characteristics and the phase-matching conditions. The single-atom harmonic source is shown in Fig. S7d-f, and the propagated harmonic fields throughout the medium are shown in Fig. S4b, d, and e. For example, there is no annular source at

20 Torr, and the phase-matched region is around $r = 20$ μm to $r = 40$ μm. With increasing gas pressure, a narrow annular source gradually forms, and the off-axis region (near $r = 50$ μm) can be phase-matched, thus supporting the generation of the Bessel-Gauss beam.

4 Spatio-temporal analysis of HHG from argon gas

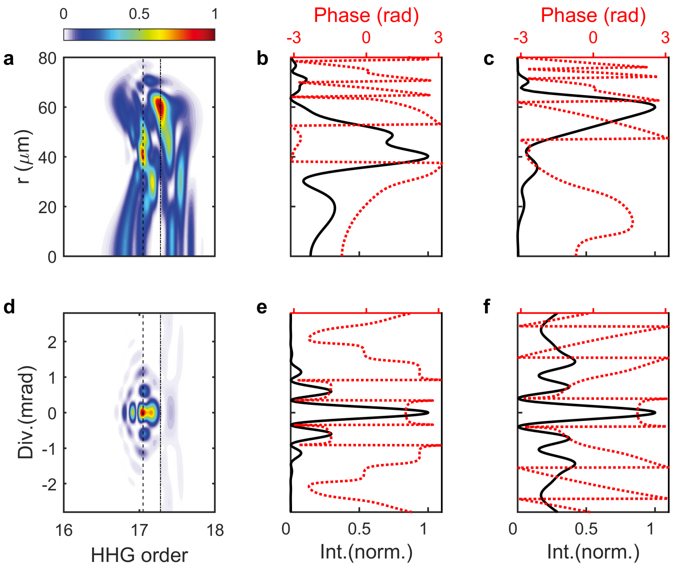

Fig. S 8 | **Spatial structure of H17 from argon gas in simulation. a,** Spatial spectral structure in the near field. **b-c,** Harmonic intensity and phase of energy slice marked by dashed and dash-dotted lines in **a**, respectively. **d**, Spatial spectral structure in the far field. **e-f,** Same as **b-c** but in the far field.

Further analysis of the propagation characteristics of the EUV beam generated from Ar gas is provided in Fig. S8. The spatial distribution of H17 within the near-field region exhibits an off-axis annular pattern, as shown in Fig. S8a. Additionally, the spatial phase behavior of the photons along two dashed lines crossing the radial axis is distinct, as illustrated in Fig. S8b, c. Consequently, the resulting beam distribution in the far-field region is shown in Fig. S8c, clearly exhibits a Bessel-like distribution. By marking two specific photon energies, both the intensity and the profile undergo significant modifications due to the diffraction phenomenon. In the near-field, the intensity of H17 is comparatively weaker than the distribution indicated by the dashed lines, which correspond to slightly higher energy levels, as demonstrated in panels (b) and (c). Notably, the spatial distribution represented in panel (b) shows a uniform phase across the radial axis, whereas the other distribution exhibits rapid phase variations. As a results, diffraction leads to a significant reconfiguration of the intensity and profile characteristics, as evident in Fig. S8e, f. Generally, the EUV Bessel-Gauss beam observed in the far-field region arises from the diffraction of an annular beam with a

diameter of approximately 40 μm, while the circular slit measures less than 10 μm, and it maintains a uniform phase profile across the circular slits.

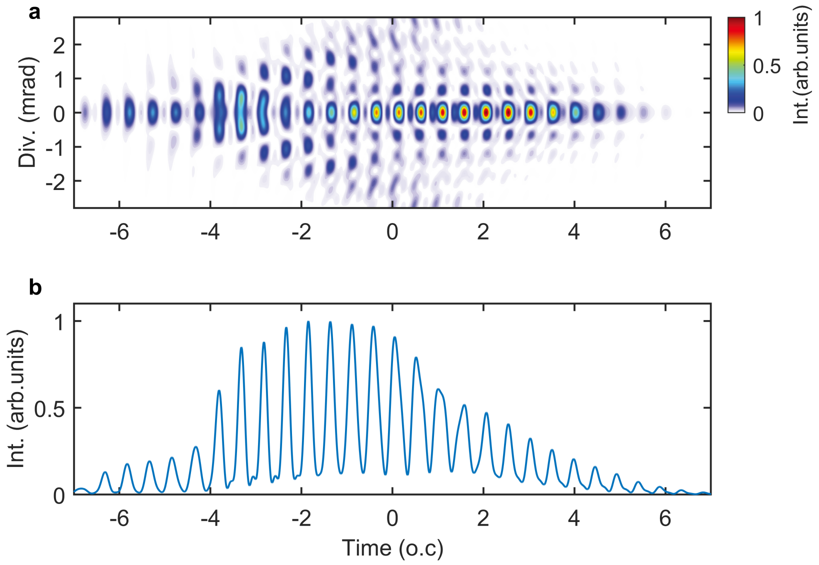

Fig. S 9 | Spatio-temporal structure of APT from Argon gas in simulation. a, Far-field APT with Bessel-like profile along radial direction synthesized by H13-H17. **b,** Integral APT in which each burst has pulse duration of 450 attoseconds.

Similar to the data presented in the main text for laser interaction with Ne gas, we also provide simulated spatial distributions and temporal evolution of HHG ranging from H11 to H17 originating from Ar gas in Fig. S3a. In this representation, distinct sub-cycle emission patterns are evident in the temporal domain, while Bessel-like profiles appear along the divergence axis. Additionally, the synthesized profiles of the attosecond pulse train (APT) in the time domain are depicted in Fig. S9. Notably, the full width at half maximum (FWHM) duration of each pulse within the APT is approximately 450 attoseconds. This observation highlights the presence of both spatio-temporal coherence in the HHG emission and the potential for its compression into an APT.

Furthermore, we apply the diffraction-free feature analysis to the entire spectral range of normal HHG under low argon gas pressure (10 Torr). As shown in Fig. S10a-c, no significant annular structure is observed in the near field, and the harmonics in the far field, as shown in Fig. S10d-f, exhibit a typical Gaussian profile. The same propagation analysis as in Fig. 6 of the main text has been applied to this spectrum. As shown in Fig. S10g and h, the beam diverges sharply with increasing propagation distance while maintaining a Gaussian profile in the cross section.

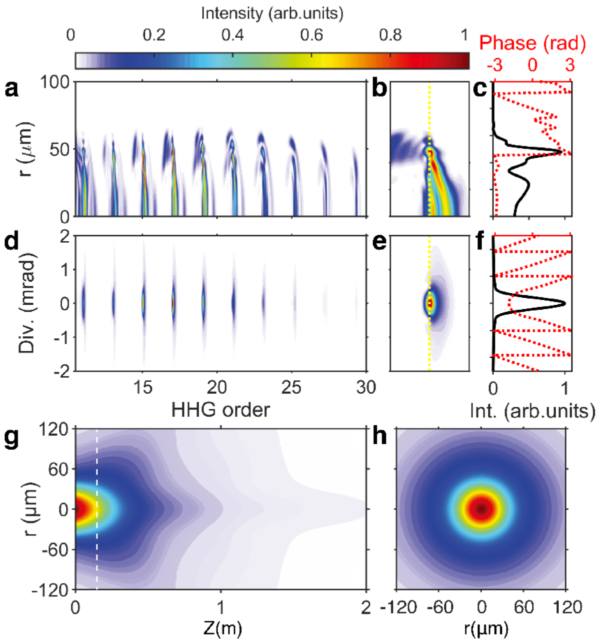

Fig. S 10 | **Simulated spatial structure of harmonics from argon at 10 Torr. a-c,** Near-field and **e-f,** far-field harmonic spectra in argon at 10 Torr. From left to right: the entire spectrum, the zoomed-in spectrum, and the line plot for the slits corresponding to the yellow dashed line. **a**, respectively. **g,**Beam profile along the propagation axis for the entire spectral range from H11 to H29, and **h,** cross section at the position of the dashed white line.

5 Validating the spatio-temporal distribution of the attosecond annular EUV beam

We further analyze the APT generated in the macroscopic gas medium at three different conditions. First, by solving coupled propagation equations of the fundamental laser and high-harmonic field, Eq. (1) and Eq. (4) in the main text, the complex harmonic field $\widetilde{E}_h^n(r, z_n, \omega)$ at the exit of gas target $z = z_n$ is obtained. Then the Fourier transform

$$\widetilde{E}_h^n(r, z_n, t) = \int_{\omega_1}^{\omega_2} \widetilde{E}_h^n(r, z_n, \omega) e^{i\omega t} d\omega \tag{1}$$

using harmonics H35-H47 is performed to obtain the spatio-temporal intensity $|\widetilde{E}_h^n(r, z_n, t)|^2$ of attosecond pulses. The spatial harmonics field in the far field $\widetilde{E}_h^f(r_f, z_f, \omega)$ is calculated with a Hankel transform (Eq. (6) in the main text). Then the coordinates r_f is transferred into divergence angle $\theta = \tan^{-1}(r_f/z_f)$, and z_f is 1000 mm in our simulation. Besides, a phase calibration according to the optical path difference $\Delta L = \sqrt{r_f^2 + z_f^2} - z_f^2$ is added as

$$\widetilde{E}_h^f(\theta, \omega) = \widetilde{E}_h^f(r_f, z_f, \omega) e^{i\omega \Delta L/c} \tag{2}$$

The attosecond pulses in the far field $|\widetilde{E}_h^f(\theta,t)|^2$ is obtained by Fourier transform of complex harmonics field $\widetilde{E}_h^f(\theta,\omega)$

$$\widetilde{E}_h^f(\theta,t) = \int_{\omega_1}^{\omega_2} \widetilde{E}_h^f(\theta,\omega)e^{i\omega t}d\omega \tag{3}$$

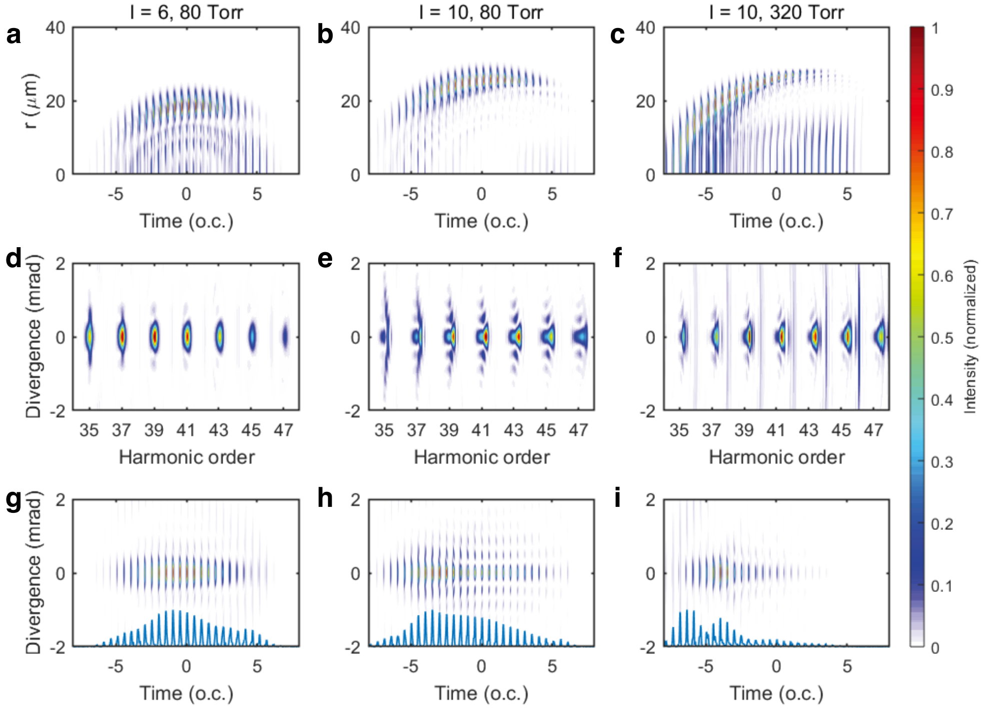

Fig. S 11 | **Theoretical spatio-temporal analysis. a-c,** Simulated attosecond pulses at the exit of gas medium generated by using harmonics of H35-H47 at different laser intensity I and gas pressure. The laser intensity is in the unit of 10^{14} W cm^{-2}. **d-f,** Spatial spectrum of harmonics in the far field with repsect to **a-c**. **g-i**, Attosecond pulses in the far field by Fourier transform of **d-f** repsectively.

Note that experimental detection lacks phase information, making it impossible to back-propagate to the near field using diffraction theory. In contrast, in numerical simulations, both near-field and far-field harmonics are treated as complex fields, and their propagation properties can be investigated using diffraction theory.

When laser intensity is 6×10^{14} W cm^{-2} and gas pressure is 80 Torr (Fig. S11a), the APT near the axis is significant despite that the major part is off-axis. As the laser intensity is increased to 10×10^{14} W cm^{-2} while maintaining the gas pressure, the APT near the axis vanishes and forms into a good annular source. After gas pressure is increased to 320 Torr in Fig. S11c, the attosecond pulses emits almost at

the leading edge and covers a wide range along the radial position. These profiles were then propagated to the far field by using the Huygens-Fresnel diffraction integral in Fig. S11d-f. And APT in the far field, as shown in Fig. S11g-i is then obtained by a Fourier transform of the spectrum. In the near field, if the harmonic has an annular distribution, then its spatial distribution transforms into a Bessel-Gauss distribution as it propagates to the far field, as demonstrated in Fig. 11e. Remarkably, this oscillatory behavior and the diminishing off-axis intensity structure agree with those observed in the experimental data.

6 Ray tracing simulation

Based on the annular source generated above, we analyzed the synthesis and detection of EUV Bessel-Gauss beams using ray tracing simulation according to the experimental setting and EUV optics. As shown in Fig. S12a-c, we first performed the ray tracing using the Gaussian-distributed EUV source (H39) with the EUV flat-field grating. We detected the source, its propagation to the grating front, and the focal plane separately. After passing through the grating, the energy of the corresponding photons in the horizontal direction is compressed, but the vertical direction remains Gaussian distributed, consistent with general harmonic measurements. For the annular EUV source shown in Fig. S12d-f, the spatial Bessel distribution before the grating is generated from the propagating diffraction, however, the Bessel distribution is retained in the vertical direction at the final probe plane after passing grating.

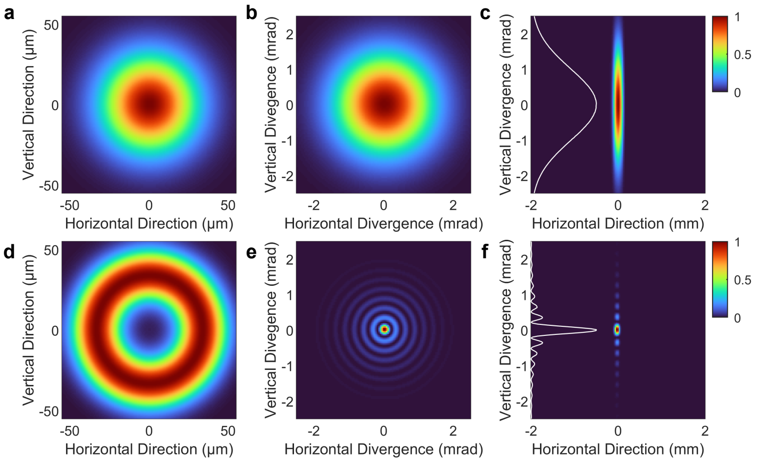

Fig. S 12 | **Beam tracing with an EUV flat-field grating. a,** EUV Source with Gauss distribution. **b,** Free propagation to the front of the grating. **c,** Captured spectra by the EUV-CCD located in the focal plane of the grating. **d-f,** Same analyses but for the annular EUV source.

To produce the EUV Bessel-Gauss beam, we designed a toroidal mirror with a grazing incidence angle of 3°, a 500 mm front focal length, and an approximate 10 km back focal length (resulting in nearly parallel light output). As shown in Fig.

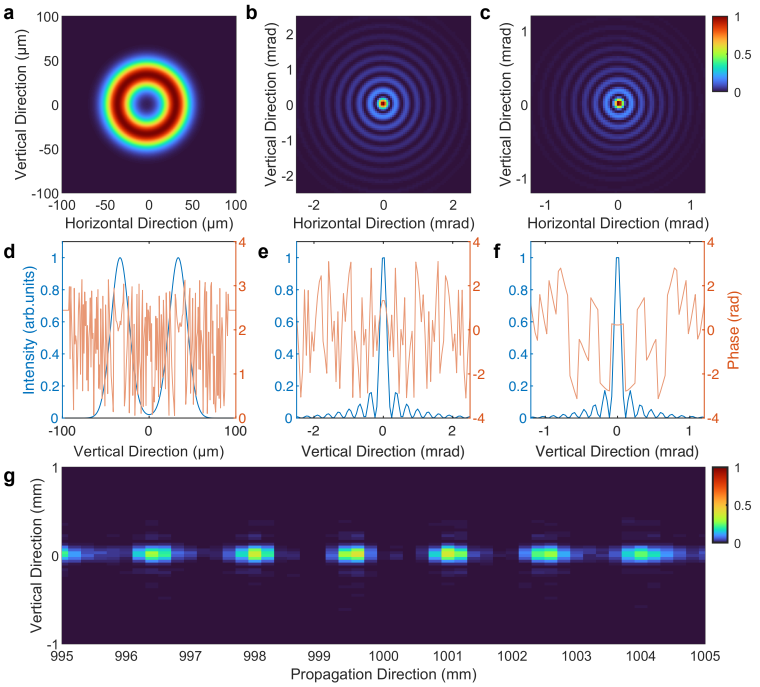

Fig. S 13 | Beam tracing with an EUV focusing element.a, Annular EUV beam source. **b,** Free propagation to the front of the toroidal mirror. **c,** 1000 mm after the toroidal mirror. **d-f,** Amplitude and phase of the vertical cross section. **g**, Intensity oscillations along the propagation axis after toroidal mirror.

S13, the annular source (a) propagates freely, forming a Bessel-like distribution (b) in front of the focusing element. After focusing, the distribution and spot size are maintained in (c), although the two differ fundamentally. As illustrated in Fig. S13d-f, the freely propagating Bessel-like beam exhibits continuous phase variation similar to the annular source. In contrast, the synthesized beam after passing through the focusing mirror exhibits a significant phase jump between the different ring structures and a pronounced intensity oscillation along the cross-section of the propagation axis, clearly indicating the formation of an EUV Bessel-Gauss beam.
